# Early adverse experiences of abuse, ventral striatum, and later life cognitive functioning

**DOI:** 10.1002/alz.087858

**Published:** 2025-01-09

**Authors:** Morgane Kuenzi, Delia A Gheorghe, Sarah Bauermeister

**Affiliations:** ^1^ University of Oxford, Oxford United Kingdom; ^2^ Transylvanian Institute of Neuroscience, Cluj‐Napoca, Cluj Romania; ^3^ Dementias Platform UK ‐ University of Oxford, Oxford United Kingdom

## Abstract

**Background:**

According to the World Health Organization, dementia is one of the leading causes of death and at least 55 million people worldwide currently have dementia. Therefore, identifying the factors that increase the risk of developing dementia, but also those that protect against it, as well as the mechanisms underlying these effects, are essential for prevention and the development of interventions.

**Method:**

Literature findings underline the detrimental effects of early adverse experiences on a variety of later‐life biopsychosocial outcomes, including brain and cognition. Adversity and its consequences may, directly and indirectly, impact cognitive functioning and lead to an increased risk of developing dementia. However, the underlying mechanisms of the association between early adversity and cognition are not yet understood.

Our research aimed to understand the direct and indirect associations between early adversity and later life cognitive impairment as well as the potential mechanisms underlying these associations. A particular focus was given to the ventral striatum given previous evidence of its association with early adversity and its involvement in reward processing.

**Result:**

Using the UK Biobank dataset, structural equation modeling (SEM) was performed.

The preliminary results highlight that the experience of early abuse is associated with a higher number of addictions. Individuals having experienced sexual abuse and emotional abuse showed slower reaction times. Addiction was associated with a smaller volume of the left ventral striatum. Interestingly, a higher volume of the left ventral striatum was associated with a faster reaction time. Finally, a significant mediation between the experience of early sexual abuse and the volume of the left ventral striatum via addiction was found.

**Conclusion:**

The preliminary results found are in line with the literature findings showing associations between adversity and addiction and between adversity and cognition. In addition, the results suggest a mediating link between sexual abuse and the left ventral striatum via addiction, highlighting the importance of addiction in this association. Although direct associations between early adversity and reaction time were found, these associations were not mediated by the volume of the left ventral striatum. These findings highlight a potential pathway by which early adversity may affect the brain.

